# Increasing Research Opportunities in Asia Amidst Dramatically Changing Epidemiologic Patterns

**DOI:** 10.2188/jea.JE20170235

**Published:** 2018-06-05

**Authors:** Naoki Kondo

**Affiliations:** Departments of Health and Social Behaviour and Health Education and Health Sociology, The University of Tokyo, Tokyo, Japan

**Keywords:** Asia, epidemiologic transition, health disparity, social determinants of health, policy

The distributions of diseases change alongside social development. For example, the concept of epidemiologic transition posits that major diseases transform from communicable to non-communicable and chronic diseases alongside economic development.^1^ The transition of major diseases also changes disease patterns across social classes. In countries where many people suffer from life-threatening absolute poverty, chronic health problems, including being overweight, are less prevalent among those who are socially vulnerable. In these countries, obesity more often afflicts the affluent population. This is because most chronic diseases, such as cancer and cardiovascular disease, occur in the late periods of life, and as people who are impoverished lack sufficient access to food and other resources, they are unlikely to live long lives. However, when countries become wealthier and absolute poverty is largely resolved, obesity afflicts the poor rather than the rich. In these countries, obesity is not a consequence of absolute poverty, but of relative poverty. That is, poverty or deprivation does not imply shortages in life-maintaining resources, but in the opportunities to make good choices, such as healthy foods, physical activities, and social engagement.^2^^,^^3^

Therefore, studies to understand transitions in the geographic and socio-demographic patterning of diseases are critically important. These studies provide essential information for national and local governments to develop public health policies. Government needs data to prioritize, make decisions with multiple stakeholders, and evaluate policies.^4^ In areas where infectious diseases dominate, the government should prioritize investing in basic social infrastructure to create hygienic environments, mitigate poverty, and stabilize political conditions.^5^ In areas where the dominant diseases are chronic, in which epidemiologic transition has already occurred, efforts to align their complex social systems and other macrosocial determinants of health to their health problems (eg, population aging, welfare protection, and controlling unhealthy behaviors) are necessary.

This issue of the *Journal of Epidemiology* includes an original contribution from Malaysia, where epidemiologic transition is currently occurring.^6^ Mariapum et al used the three waves (1996, 2006, and 2011) of the Malaysian National Health and Morbidity Survey to effectively describe temporal trends in the multiethnic nation in terms of income-based inequality in being overweight and obesity by gender, area, and ethnicity. The findings can be interpreted as follows. In East Malaysia, epidemiologic transition did not occur in 2011. For both men and women, overweight/obesity is less prevalent among the poor than the rich. In Peninsular Malaysia, the trends are similar for men, but women likely began transitioning from 1996 to 2011, during which time the prevalence of overweight increased more among low-income households than high-income ones, especially after 2006. Observing the trends by ethnicity, for Chinese Malaysians, the wealthiest of the three ethnicities, women demonstrated a low-income/high overweight relationship throughout the period observed, whereas for Malay Malaysians, the pre-transition low-income/less overweight pattern was indicated. This observation provides strong messages for the Malaysian government regarding where and whom they should prioritize in their public policies to address issues pertaining to health disparity. This study could ignite debates on the potential reasons for the incoherent processes of epidemiologic transition by area, ethnicity, and gender, and provide future research questions and interesting perspectives in the fields of social epidemiology, health sociology, and demographics. For example, inconsistent patterns may be due to differences in material conditions, cultures, and social norms (eg, for body image) among subpopulations.

Asia is an exciting place in which to study society and health. Asia comprises countries in various phases of economic development, covering 99% of the range of variations in country per capita income (from North Korea to Macao, China). Asia also includes 81% of the range of variations in life expectancy worldwide (from Afghanistan to Japan).^7^ Among the 10 most populous countries, six are in Asia (ie, China, India, Indonesia, Pakistan, Bangladesh, and Japan). Excepting Japan, these highly populated regions are all middle-income countries, wherein we witness rapid economic development and the associated social changes. As such, epidemiologic transition may be occurring in many regions of these countries.

**Figure 1.**
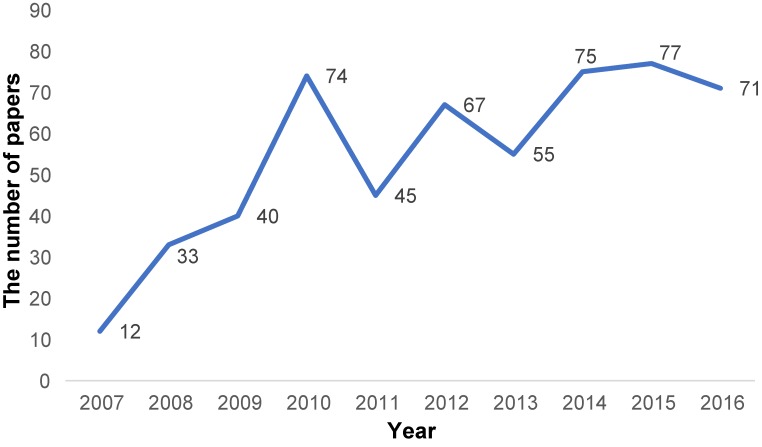
Number of papers from Asian countries using nationwide surveys published in 11 major epidemiology journals in PubMed

Opportunities for epidemiologic study in Asia are increasing. I conducted a brief PubMed paper search on August 31, 2017, and counted peer-reviewed original articles using national or nationwide surveys from the Asian region. I used the keywords “national survey” or “national data” or “nationwide,” and searched the papers published during the last 10 years (until the end of 2016) in 11 major epidemiology journals. I identified 597 papers in total, a number that has increased over time (Figure 1). Especially, many contributions are from Taiwan and South Korea, where national data infrastructure is rapidly developing. In the *Journal of Epidemiology*, our paper search found 29 papers from Taiwan, 20 from South Korea, 4 from China, 4 from Thailand, and 1 each from Iran, Indonesia, Mongolia, and the United Arab Emirates (excluding 108 papers from Japan).

Researchers should expect the rapid improvement of data availability in Asian countries. As an editorial member of the *Journal of Epidemiology*, I welcome more papers based on good data and analytic strategies from Asian countries. Given the many high-quality papers from South Korea and Taiwan, and interesting new evidence from Malaysia in this issue, researchers in other Asian countries should seek further opportunities to establish and develop data infrastructure. A regular data gathering framework provides further opportunities for natural experimental studies, making it possible to formally evaluate the impacts of social changes on health when influential policies or phenomena occur, such as natural disasters, economic crises, and conflicts.^8^^–^^10^
